# Adverse events associated with continuous interscalene block administered using the catheter-over-needle method: a retrospective analysis

**DOI:** 10.1186/s12871-019-0873-9

**Published:** 2019-10-28

**Authors:** Meishu Tanijima, Kenichi Takechi, Kazuo Nakanishi, Toshihiro Yorozuya

**Affiliations:** 1 Ehime Prefectural Central Hospital, 83 kasugachou, Matsuyama City, Ehime Japan; 2 Matsuyama Red Cross Hospital, 1 Bunkyochou, Matsuyama City, Ehime Japan; 3 Ehime Prefectural Imabari Hospital, 4-5-5 Ishiichou, Imabari City, Ehime Japan; 4 Department of Anesthesia and Perioperative Medicine, Ehime University Graduate School of Medicine, 454 Shitsukawa, Toon City, Ehime Japan

**Keywords:** Continuous interscalene block, Catheter-over-needle method, Adverse event

## Abstract

**Background:**

Continuous interscalene block is widely used for pain management in shoulder surgery. However, continuous interscalene block performed using the catheter-through-needle method is reportedly associated with adverse events such as pericatheter leakage of the local anesthetic, phrenic nerve paralysis, and hoarseness. Because we expected that the catheter-over-needle method would reduce these adverse events, we examined cases in which continuous interscalene block was performed using the catheter-over-needle method to determine what adverse events occurred and when.

**Methods:**

We retrospectively reviewed the anesthesia and medical records of adult patients who underwent catheter insertion to receive a continuous interscalene block performed using the catheter-over-needle method at our hospital from July 2015 to July 2017.

**Results:**

During the surveillance period, 122 adult patients underwent catheter insertion to receive a continuous interscalene block administered using the catheter-over-needle method. No case of pericatheter local anesthetic leakage was observed. Adverse events, such as dyspnea, hoarseness, insufficient anesthetic effect, dizziness, cough reflex during drinking, or ptosis, were observed in 42 patients (34.4%; 95% confidence interval 26–42.7). Most of the adverse events occurred on postoperative day 2. The median time between surgery and the onset of adverse events was 28.5 h.

**Conclusions:**

The catheter-over-needle method may prevent the pericatheter leakage of the local anesthetic. However, adverse events occurred in more than one-third of the patients. During continuous interscalene block, patients must be carefully observed for adverse events, especially on postoperative day 2.

**Trial registration:**

This study was registered at the UMIN Clinical Trials Registry on August 13th, 2019 (UMIN000037673).

## Background

Shoulder surgery is known to be associated with severe pain not only postoperatively but also during rehabilitation. Continuous interscalene block, incorporating the basal infusion of a local anesthetic and patient-controlled boluses, is one of the most effective and frequently used analgesic techniques after both major and minor shoulder surgeries [1–4]. Many authors have supported the efficacy and safety of continuous interscalene block in ambulatory patients [5–9]. These reports have focused on the adverse events associated with continuous interscalene block performed using the catheter-through-needle method [10, 11]. In this method, the outer diameter of the catheter is smaller than the initial needle-punctured hole. Therefore, there is a potential for pericatheter leakage of the local anesthetic and perineural catheter dislocation.

Conversely, in the catheter-over-needle method, the catheter fits tightly in the puncture hole, which reportedly reduces the incidence of pericatheter local anesthetic leakage and perineural catheter dislocation [12, 13]. However, the incidence of adverse events associated with continuous interscalene block performed using the catheter-over-needle method has not been fully investigated, with the only adverse events reported being the pericatheter leakage of the local anesthetic and perineural dislocation of the catheter. Therefore, the purpose of this study was to determine the adverse events that occurred using continuous interscalene block performed using this method and the time of occurrence of these adverse events.

## Methods

After obtaining institutional board approval (registration number 29–3), we reviewed the anesthesia and medical records of all adult patients who underwent catheter insertion to receive continuous interscalane block administered by anesthesiologists using the catheter-over-needle method at the Ehime Prefectural Imabari Hospital from July 2015 to July 2017. We excluded patients in whom the catheter was inserted by orthopedists. We collected data on patient characteristics, the incidence of pericatheter leakage of the local anesthetic, insufficient anesthetic effect (postoperative pain numerical rating scale score ≥ 3), and symptoms suggestive of a neurological complication (e.g., dyspnea, hoarseness, dizziness, cough reflex during drinking, and ptosis) from the time of insertion of the catheter to its removal. Informed consent to perform continuous interscalane block was obtained from all patients. The study was registered with the UMIN Clinical Trials Registry (UMIN000037673).

### Catheter insertion procedure

In all patients, the catheter was inserted just before the induction of general anesthesia in the operating room, without preoperative sedation or premedication. Each patient was placed in the supine position; the arm on the side to be operated on was at the patient’s side, and the head was turned slightly to the contralateral side. A Sonosite M-Turbo ultrasound system with a 6 to 13 MHz linear probe (Sonosite Inc., Bothell, Wash.) was used. After preoperative scanning around the interscalene brachial plexus for correct location of catheter placement, the area was sterilized, and continuous interscalene block was performed using the Contiplex® C Catheter-Over-Needle Continuous Nerve Block Set (B.Braun Melsungen AG, Melsungen, Germany) under in-plane ultrasound guidance without nerve stimulation via a lateral approach. The catheter tip was passed through and placed between C5 and C6 in the brachial plexus. After injecting 2 to 3 mL of lidocaine to confirm that the catheter tip was correctly placed for the administration of anesthetic, 10 mL of 0.75% ropivacaine was administered in a bolus through the catheter under ultrasound visualization for surgical anesthesia. The catheter insertion site was sealed with topical medical cyanoacrylate glue (Dermabond; Ethicon, San Angelo, Tex.) and draped with a clear film (3 M Tegaderm™ Film, 6 cm × 7 cm; 3 M Health Care, St. Paul, Minn.).

### Perioperative management

In all patients, a standard anesthetic technique was used, wherein the non-invasive arterial blood pressure, electrocardiogram, and oxygen saturation were routinely monitored in the operating room. General anesthesia was induced with propofol (1.5–2 mg/kg), remifentanil (0.15–0.3 μg/kg/min), and rocuronium bromide (0.8 mg/kg) and maintained with sevoflurane (1.5–2%) and remifentanil (0.15–0.3 μg/kg/min). The airway was secured using an LMA ProSeal™ laryngeal mask (Teleflex, Westmeath, Ireland). After a patient’s emergence from anesthesia, the catheter was connected to an elastomeric pump (Coopdech® Balloonjector®: Daiken Medical Co., Ltd. Osaka, Japan), and the patient received an infusion of 0.2% ropivacaine at 4 or 6 mL/h and was given access to a patient-controlled system to receive a bolus of 5 mL with lock-out time 30 min. Drugs used for postoperative analgesia included 50 mg of flurbiprofen axetil and 1000 mg of acetaminophen administered intravenously, or delivered via local anesthetic bolus, depending on patients’ and nurses’ preferences. If adverse events occurred, the local anesthetic flow was stopped or decreased until the event subsided, and then the flow was resumed.

From July 2015 to May 2016, all patients received 0.2% ropivacaine at 4 mL/h until postoperative day 1 and then 2 mL/h until catheter removal. From June 2016 to July 2017, all patients received 0.2% ropivacaine at 6 mL/h until postoperative day 1, 4 mL/h until postoperative day 2, and 2 mL/h until catheter removal.

### Statistical analysis

All statistical analyses were performed using R 3.1.2 software. Continuous variables are presented as means and standard deviations. Categorical variables were compared using Fisher’s exact test or χ^2^ test as appropriate; continuous variables were compared using Student’s *t*-test. All the reported *p* values were two-sided, and a *p* value of < 0.05 was considered significant.

## Results

During the observation period, 122 patients received continuous interscalene block using the catheter-over-needle method. Of these patients, 102 (83.6%) underwent arthroscopic surgery and the remaining underwent open surgery. In the 102 patients, local anesthetic infusion was continued over postoperative day 4; furthermore, in 80 of them, it was continued until postoperative day 7. In the remaining 20 patients, the infusion was stopped before postoperative day 4 because the patients did not wish to continue the infusion or the catheter was accidentally removed. Table 1 shows the characteristics of the study population.

**Table 1:** Characteristics of the study population

Sex, male/female, n	70/52
Age (years), mean ± SD	64 ± 16
Height (cm), mean ± SD	159 ± 9
Weight (kg), mean ± SD	63 ± 12

SD: standard deviation

### Adverse events and onset time

No pericatheter leakage of the local anesthetic was observed. The anesthetic effect was insufficient in 13 cases (10.7%); other adverse events included dyspnea in 4 (3.3%), hoarseness in 12 (9.8%), dizziness in 15 (12.3%), cough reflex during drinking in 3 (2.5%), and ptosis in 2 (1.6%) cases. Overall, adverse events were observed in 42 cases (34.4%).Table 2 lists the adverse events and the median time at onset.

**Table 2:** Adverse events

Adverse event	n (%), (95%CI)	Onset time (h), median
Catheter leakage	0	
Insufficient effect case	13 (10.7), (5.0–16.3)	
Dyspnea	4 (3.3), (−0.4–6.9)	30.8
Hoarseness	12 (9.8), (4.3–15.3)	29.5
Dizziness	15 (12.3%), (6.3–18.2)	26.5
Cough reflex during drinking	3 (2.5), (−0.9–5.8)	28.5
Ptosis	2 (1.6), (−1.3–4.6)	40
Total	42 (34.4), (26–42.7)	28.5

95% CI: 95% confidence interval

Almost all symptoms were mild and well tolerated. More than two adverse events were observed in seven cases. Most of the adverse events occurred on postoperative day 2. The median time from the start of the continuous infusion until the onset of the adverse event was 28.5 h. Table 3 shows the differences between patients with different rates of local anesthetic flow. There were no differences between these groups in the incidence of dyspnea (*P* = 0.867), hoarseness (*P* = 0.757), dizziness (*P* = 0.624), cough reflex during drinking (*P* = 0.106), and ptosis (*P* = 0.189).

**Table 3:** Patient characteristics and outcomes with different rate of anesthetic infusion

	4 mL/h (*n* = 56)	6 mL/h (*n* = 66)	*P* value
Sex, male/female, n	34/22	36/30	0.492
Age (years), mean ± SD	59 ± 18	67 ± 14	< 0.05
Height (cm), mean ± SD	161 ± 9	157 ± 9	< 0.05
Weight (kg), mean ± SD	66 ± 13	61 ± 9	< 0.05
Dyspnea, n	2	2	0.867
Hoarseness, n	5	7	0.757
Dizziness, n	6	9	0.624
Cough reflex during drinking, n	0	3	0.106
Ptosis, n	0	2	0.189

SD: standard deviation

## Discussion

In this study, continuous interscalene block administered using the catheter-over-needle method prevented the pericatheter leakage of the local anesthetic. The incidences of adverse events observed using this method in the present study seem to be lower than those reported using the catheter-through-needle method (catheter leakage, 8%; hoarseness, 13%; dyspnea, 27%; and dysphagia, 7%) in a previous study [10].

The catheter-over-needle method reduces the pericatheter leakage of the local anesthetic [13]. In addition, we used medical glue at the catheter insertion site that reportedly prevents such leakage during continuous perineural infusion [14]. These two steps completely prevented the pericatheter leakage of the local anesthetic in our patients.

Adverse symptoms suggestive of neurological complications (dyspnea, hoarseness, cough reflex during drinking, and ptosis) were observed in 19 cases. The catheter-over-needle method under in-plane ultrasound guidance enabled us to place the catheter tip in the interscalene space in the brachial plexus under direct visualization and with tight fixation [15]. Such placement helped prevent perineural catheter tip dislocation and may have reduced the incidence of unintended nerve block caused by inappropriate catheter tip position. Thus, the incidence of adverse events involving continuous interscalene block can be expected to be lower using the catheter-over-needle method than using the catheter-through-needle method. However, even if the catheter tip is in the correct position, local anesthetics may spread to the anterior part of the anterior scalene muscle, resulting in phrenic nerve block and dyspnea [16]. Similarly, when local anesthetics spread to the anterior part of the anterior scalene muscle through the prevertebral layer of the deep cervical fascia, the recurrent laryngeal nerve is blocked, resulting in hoarseness or cough reflex during drinking, or both [17]. Moreover, when local anesthetics spread to the cervical sympathetic ganglion, ptosis is observed as part of Horner’s syndrome [18]. Sympathetic block or local anesthetic systemic toxicity causes dizziness. We did not observe any significant differences in the incidence of adverse events according to the local anesthetic flow rate. Anatomical differences among patients (e.g., variations in nerve position or differences in loose connective tissue around the interscalene space) may account for the extent of neurological complications.

All the symptoms observed in our study patients were mild and subsided after a decrease in or temporary cessation of the local anesthetic flow. Most of the adverse events occurred on postoperative day 2, and the median time from the start of continuous infusion to the onset of the adverse event was 28.5 h. Thus, patients must be observed carefully after surgery, especially on postoperative day 2.

The possible reasons for insufficient anesthetic effect in 13 cases even after an effective interscalene block are as follows: the interscalene block cannot cover the intercostal brachial nerve that innervates areas of the axilla, the lateral aspect of the chest, and the medial aspect of the arm. To cover the incision of the intercostal brachial nerve area, serratus plane block or local anesthetic infiltration into the incision site may be performed [19]. Intraoperative and postoperative routine protocols for systemic and structured oral analgesia are also rescuing the insufficient effect case.

This study had some limitations. It was a retrospective study, and the sample size was smaller than that of the other study with which comparisons were made. Another prospective study is needed to compare the incidence of adverse events between the catheter-over-needle method and the catheter-through-needle method; however, our findings suggest that adverse events were less frequent with the former than with the latter.

## Conclusion

In conclusion, more than one-third of patients experienced adverse events during the continuous interscalene block even after using the catheter-over-needle method. Therefore, patients must be observed carefully during continuous interscalene block, especially on postoperative day 2, and patient education about adverse events is crucial.

## Data Availability

All data generated or analyzed during the current study are included in this article and are available from the corresponding author on reasonable request.

## Abbreviations

CI: confidence interval
SD: standard deviation
